# Biometal Dyshomeostasis in Olfactory Mucosa of Alzheimer’s Disease Patients

**DOI:** 10.3390/ijms23084123

**Published:** 2022-04-08

**Authors:** Riikka Lampinen, Veronika Górová, Simone Avesani, Jeffrey R. Liddell, Elina Penttilä, Táňa Závodná, Zdeněk Krejčík, Juha-Matti Lehtola, Toni Saari, Juho Kalapudas, Sanna Hannonen, Heikki Löppönen, Jan Topinka, Anne M. Koivisto, Anthony R. White, Rosalba Giugno, Katja M. Kanninen

**Affiliations:** 1 A. I. Virtanen Institute for Molecular Sciences, University of Eastern Finland, 70210 Kuopio, Finland; riikka.lampinen@uef.fi (R.L.); veronika.gorova@uef.fi (V.G.); 2 Department of Computer Science, University of Verona, 37134 Verona, Italy; simone.avesani@univr.it (S.A.); rosalba.giugno@univr.it (R.G.); 3 Department of Biochemistry and Pharmacology, The University of Melbourne, Melbourne, VIC 3010, Australia; jliddell@unimelb.edu.au; 4 Department of Otorhinolaryngology, University of Eastern Finland and Kuopio University Hospital, 70210 Kuopio, Finland; elina.penttila@kuh.fi (E.P.); heikki.lopponen@kuh.fi (H.L.); 5 Department of Genetic Toxicology and Epigenetics, Institute of Experimental Medicine of the Czech Academy of Sciences, Videnska 1083, 142 20 Prague, Czech Republic; tana.zavodna@iem.cas.cz (T.Z.); zdenek.krejcik@iem.cas.cz (Z.K.); jan.topinka@iem.cas.cz (J.T.); 6 Brain Research Unit, Department of Neurology, School of Medicine, University of Eastern Finland, 70210 Kuopio, Finland; juha-matti.lehtola@uef.fi (J.-M.L.); toni.saari@uef.fi (T.S.); juho.kalapudas@uef.fi (J.K.); sanna.hannonen@kuh.fi (S.H.); anne.koivisto@kuh.fi (A.M.K.); 7 Department of Neurology, NeuroCentre, Kuopio University Hospital, 70210 Kuopio, Finland; 8 Department of Neurology and Geriatrics, Helsinki University Hospital and Neurosciences, Faculty of Medicine, University of Helsinki, 00014 Helsinki, Finland; 9 Department of Cell and Molecular Biology, Mental Health Program, QIMR Berghofer Medical Research Institute, Herston, QLD 4006, Australia; tony.white@qimrberghofer.edu.au

**Keywords:** Alzheimer’s disease, olfactory mucosa cells, biometals, zinc, calcium, sodium, alpha-2-macroglobulin, olfactory dysfunction

## Abstract

Olfactory function, orchestrated by the cells of the olfactory mucosa at the rooftop of the nasal cavity, is disturbed early in the pathogenesis of Alzheimer’s disease (AD). Biometals including zinc and calcium are known to be important for sense of smell and to be altered in the brains of AD patients. Little is known about elemental homeostasis in the AD patient olfactory mucosa. Here we aimed to assess whether the disease-related alterations to biometal homeostasis observed in the brain are also reflected in the olfactory mucosa. We applied RNA sequencing to discover gene expression changes related to metals in olfactory mucosal cells of cognitively healthy controls, individuals with mild cognitive impairment and AD patients, and performed analysis of the elemental content to determine metal levels. Results demonstrate that the levels of zinc, calcium and sodium are increased in the AD olfactory mucosa concomitantly with alterations to 17 genes related to metal-ion binding or metal-related function of the protein product. A significant elevation in alpha-2-macroglobulin, a known metal-binding biomarker correlated with brain disease burden, was observed on the gene and protein levels in the olfactory mucosa cells of AD patients. These data demonstrate that the olfactory mucosa cells derived from AD patients recapitulate certain impairments of biometal homeostasis observed in the brains of patients.

## 1. Introduction

Alzheimer’s disease (AD) is a progressive neurodegenerative disorder, and the most common cause of dementia. Genetic, lifestyle and environmental factors are involved in the pathogenesis of AD, so the exact disease mechanisms remain incompletely understood. The late onset AD (LOAD) is a multifactorial disease caused by lifestyle, environmental factors and a poor genetic component but it is important because it is responsible for more than 90% of the cases of AD (reviews [1,2]). The pathological proteins associated with AD begin to accumulate in the brain already decades before the patients report any clinical symptoms (review [3]). A precursor disorder named mild cognitive impairment (MCI) is thought to be an early form of AD in a subset of individuals (review [1]). Interestingly, one of the earliest symptoms of AD, which is also associated with certain other neurodegenerative diseases, is olfactory dysfunction [4,5] (meta-analysis [6]). We have shown earlier that the cells of the olfactory mucosa (OM), situated at the rooftop of the nasal cavity, show several pathological features observed in AD brains, including increased secretion of amyloid-beta (Aβ_1–42_) [7].

Biometal homeostasis is crucial for tissue function, as metals are needed for cellular signaling and serve as co-factors for a multitude of enzymes. Dyshomeostasis of biometals is a commonly observed feature of neurodegeneration and evident in AD affected brains (review [8]). Alterations of several elements, including copper, zinc and iron are commonly observed in postmortem brains, although at times, contradictory results have been presented with a subset of studies showing elevations, and others describing reductions in particular elements. Interestingly, some changes in metals are reported to occur in the olfactory bulb of AD patients [9], and the levels of certain metals are high in the olfactory bulbs in comparison to other areas of the central nervous system [10]. In addition, changes in the levels of metal transporters and metal binding proteins are also described in the AD brain (review [11]). While the exact reason for altered metal homeostasis in AD remains unknown, it has been suggested to be linked to metal-catalyzed oxidation relating to transition metal ions that may lead to mitochondrial dysfunction (review [8]) and impaired metal buffering by proteins including the metallothioneins (reviewed in [8,12,13]).

In the present study, we addressed the question of whether alterations to biometal homeostasis observed in AD patient brains are also reflected in the OM. We applied RNA sequencing to discover alterations to metal-linked genes and pathways, which yielded a total of 17 genes related to metal-ion binding or with metal-related function that were significantly altered between AD and control OM cells. MCI OM cells displayed, respectively, a total of 14 metal-related and differentially expressed genes. Gene Ontology enrichment analysis of the genes significantly differently expressed between AD and control OM cells highlighted calcium ion binding as enriched molecular function of these genes. Alterations in the gene expression of alpha-2-macroglubulin, *A2M*, was further validated on the protein level and shown to be significantly increased in AD OM cells. Further support for biometal imbalance in the AD OM cells was provided by analysis of the elemental content by inductively coupled plasma mass spectrometry (ICP-MS), implicating an increase in the cellular levels of sodium, calcium and zinc in AD OM cells.

## 2. Results

### 2.1. Transcriptomic Analysis of Metal-Related Gene Alterations in AD OM Cells

**Figure 1:**
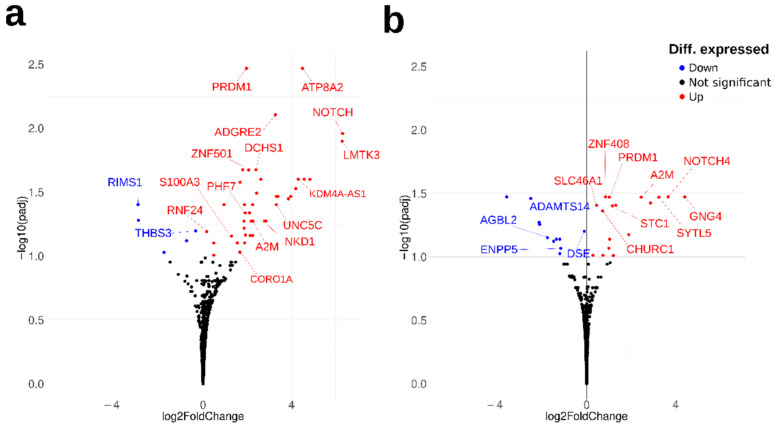
Transcriptomic metal-related gene alterations in AD) and MCI olfactory mucosa (OM) cells. The volcano plot visualizes the differentially expressed genes (DEGs) between the OM cells derived from cognitively healthy controls and patients with (**a**) Alzheimer’s disease (AD) or (**b**) mild cognitive impairment (MCI). The significant DEGs upregulated in AD OM cells are visualized in red (positive fold change) and the significantly downregulated DEGs in blue (negative fold change). The metal-related DEGs are highlighted.

To assess whether biometal imbalance that is commonly observed in AD brains is also found in the OM cells of patients with AD or MCI, we first applied total RNA sequencing to OM cells (Table A1). Sequencing revealed a total of 47 differentially expressed genes (DEGs) between the AD and control OM cells (FDR < 0.1), of which 17 were related to metal-ion binding or metal-related function of the protein product between the cognitively healthy controls and AD patients (Figure 1a, Table 1 and Table S1). The majority of the DEGs between AD and controls were related to metal-binding of zinc, calcium or magnesium. Most of the DEGs were upregulated in AD OM cells, whilst only *RIMS1* and *THBS3* were significantly downregulated in AD OM cells. Gene Ontology (GO) enrichment analysis of the DEGs suggested calcium ion binding (GO:0005509) as a significantly (*p* = 0.022) enriched molecular function among the 47 DEGs between AD and control OM cells.

When comparing the gene expression of MCI and control OM cells, a total of 28 significantly differentially expressed genes (FDR < 0.1) were found (Table S2). A total of 14 of these DEGs between MCI and control OM cells were found to have metal-related functions (Figure 1b, Table 2). Similarly to AD OM cells, the metal-related functions of the DEGs between MCI and OM cells were associated mainly to metal-binding of zinc, calcium and magnesium. Four genes (*NOTCH4*, *AC253536.3*, *A2M* and *PRMD1*) were upregulated both in AD and MCI OM cells compared to the controls (Tables S1 and S2), with *NOTCH4*, *A2M* and *PRMD1* having metal-related functions (Figure 1, Table 1 and Table 2).

### 2.2. Expression of Alpha-2-Macroglobulin Is Altered in AD OM Cells

**Figure 2:**
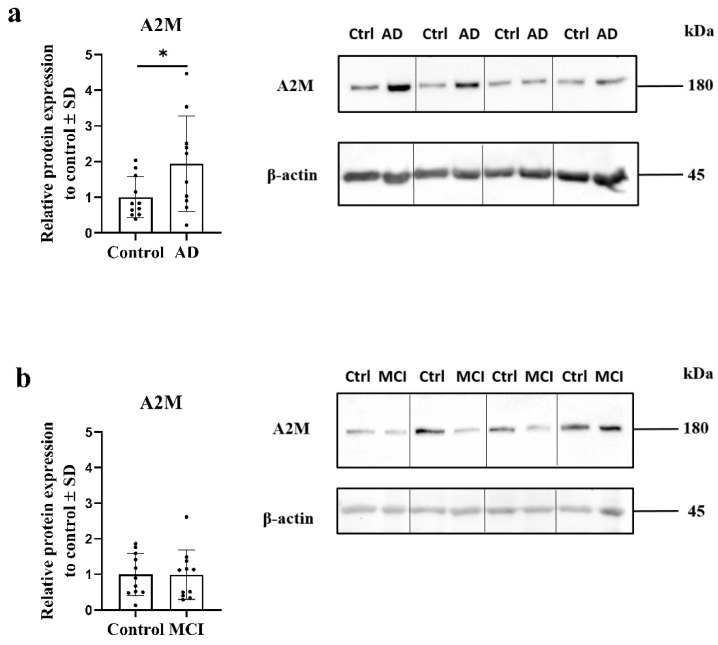
Western blot of A2M and β-actin in (**a**) AD olfactory mucosa (OM) cells and (**b**) MCI OM cells and the analysis of band intensities normalized to β-actin. All data are presented as mean ± SD. (**a**,**b**) *n* = 11 control lines, (**a**) *n* = 10 Alzheimer’s disease (AD) lines, (**b**) *n* = 11 mild cognitive impairment (MCI) lines. Outliers within the data groups were tested with iterative Grubb’s method (alpha 0.05). Unpaired two-tailed *t*-test. * *p* ≤ 0.05.

Of particular interest, both the AD and MCI OM cells were, via RNA sequencing, shown to express significantly higher levels of alpha-2-macroglobulin (A2M), encoded by *A2M*, than the cells derived from cognitively healthy individuals (Table 1 and Table 2). Previous reports have demonstrated the levels of A2M to also be increased in the brains [14] and plasma of AD patients (systematic review [15]). Enrichment analysis of the DEGs between AD and control OM cells with pathfindR, a tool also utilizing protein–protein interaction data for enrichment [16], revealed the GO term for serine-type endopeptidase inhibitor activity (GO:0004867) to be significantly enriched. DEG associated with this particular GO term was *A2M* (*p* = 0.041). A2M belongs to the LRP1–A2M–annexin VI complex, which was suggested as a significantly enriched protein complex both in AD and MCI OM cells compared to controls based on the functional analysis of the DEGs using the CORUM (the comprehensive resource of mammalian protein complexes) database (CORUM:2710, for AD *p* = 0.012, for MCI *p* = 0.032). Therefore, we next applied Western blotting to determine whether the upregulation of *A2M* by AD OM cells is also evident at the protein level. Western blotting revealed that the AD OM cells express significantly increased levels of the A2M protein when compared to the cells derived from cognitively healthy controls (*p* ≤ 0.05, Figure 2a). Levels of A2M protein were unaltered in individuals with MCI (Figure 2b).

### 2.3. Elemental Content in AD OM Cells

Since the expression of several metal-related genes were altered in AD OM cells, and the protein level of A2M was significantly altered in AD, we next measured the elemental content of Na, Mg, P, K, Ca, Mn, Fe, Cu, Zn, Se, Rb and Co using ICP-MS in AD and control OM cells (Table 3 and Table A2).

The total levels of sodium (*p* ≤ 0.01), calcium (*p* ≤ 0.05) and zinc (*p* ≤ 0.05) were significantly increased in AD OM cells, whilst the other measured elements were not significantly altered between the healthy and AD cells. The levels of Se, Rb and Co were below the detection limit of the ICP-MS measurement.

## 3. Discussion

Biometal dyshomeostasis is a well characterized feature of the AD brain and plasma, which is related to both onset and progression of the disease. Here, we show for the first time that cells of the OM, central for sense of smell, and in direct contact with the brain, also display disease-related alterations in both biometal levels, and in the expression of several metal-related genes. Specifically, we demonstrate by RNA sequencing the alteration of 17 metal-related genes between AD and control OM cells, out of which four—*NOTCH4*, *CORO1A*, *ATP8A2* and *RIMS1*—have been previously associated with AD or amyloid related cognitive decline [17,18,19,20,21]. We also report increases in the levels of intracellular zinc, sodium and calcium in OM cells derived from patients with AD. Concomitantly, we demonstrate the protein level of A2M to be significantly upregulated in AD OM cells.

Literature spanning decades of work has provided important insight into pathological mechanisms of AD, and the involvement of metals in these processes. However, the presence of conflicting reports on AD-associated changes to metal levels in the brains of patients makes it difficult to draw conclusions on the exact role of specific metals in AD pathogenesis. For example, zinc levels are reported to be increased, reduced or unchanged in the AD brain (review [22]), after the first hypothesis of the involvement of zinc in dementia by Burnet in 1981 [23]. Interestingly, increases in zinc have been previously implicated in AD in regions of the brain involved in the olfactory system [9]. Further complexity arises from the observations that subcellular alterations, or mis-localization of metal ions has also been reported (review [24]). These discrepancies could be partly explained by different experimental systems, post-mortem delay times and different analytical methods. However, imbalance or mis-localization of zinc is clearly harmful as zinc is one of the most abundant essential trace metals, the levels of which are tightly controlled. Most of the zinc in the body is bound by proteins, for which it is essential in terms of enzymatic activity or structural stability. Here, we show an elevation in intracellular zinc levels in the OM cells derived from AD patients. We also describe alterations to several genes that are bound or activated by zinc, seven between AD and control OM cells and eight between MCI and controls, including zinc finger protein coding genes. Furthermore, our previously published single cell RNA sequencing data of OM cells derived from patients with AD and their controls revealed alterations to genes that encode metallothioneins, including *MT2A* (metallothionein 2A) and *MT1X* (metallothionein 1X) especially in fibroblast/stromal-like cells [7]. The key functions of metallothioneins are to maintain the cellular metal homeostasis, and act against increased amounts of reactive oxygen species (ROS) in the cell or after stimuli of heavy metals (review [25]). Both of the metallothioneins MT1X and MT2A can bind zinc (UniProt 16.2.2022). Interestingly, astrocytes treated with zinc display increased levels of glial fibrillary acidic protein (GFAP), a marker for astrocyte activation/reactive astrogliosis [26], suggesting that tight regulation of zinc levels is essential for the normal functioning of cells. Similarly to our findings, high levels of zinc are also reported in another brain-connected sensory organ of the body, in the deposits in the eye in patients with age-related macular degeneration (AMD) [27]. AMD is a neurodegenerative disease of the retina with multiple clinical and pathological similarities to AD (review [28]). Taken together, our data supports the previously published reports on brain-related alterations in zinc balance that occurs in AD and suggests that also other tissues beyond the brain suffer from impaired zinc homeostasis in this disease.

Dysregulation of intracellular calcium is a well-described feature of the AD brain. Similar to zinc, calcium levels are increased in the vicinity of Aβ plaques, and several studies also report increases in intracellular calcium levels of brain cells (review [29]). In line with these reports, our results demonstrate that intracellular calcium levels are elevated in AD OM cells. Interestingly, metals have a key role in olfaction [30], and prior literature suggests that metal ions are transported from the olfactory mucosa to the brain (review [31]). Studies in mice have demonstrated that intranasal administration of zinc sulfate results in transient anosmia [32], and the administration of inhalational forms of zinc in humans can cause hyposmia or anosmia [33]. It is believed that zinc can block the ion channels that facilitate signal transduction in the olfactory mucosa. In addition, reducing intranasal calcium can improve olfaction in patients with smell impairments [34]. Olfactory dysfunction is an early sign of AD-associated neurodegeneration [4,5,6], and here we show that cells of the OM of AD patients have significant increases in zinc and calcium levels, and alterations in genes associated with metal-related processes. In further studies, it would be interesting to investigate in detail whether the changes in the OM cells are connected to olfactory dysfunction reported by a subset of AD patients.

Changes in the dynamic balance of metal ions are linked to the deposition of beta-amyloid and hyperphosphorylation of tau protein in the AD brain [35,36]. For example, zinc ions are associated with the processing of the amyloid precursor protein (APP), the precursor of beta-amyloid (Aβ) [37], and calcium elevation can promote Aβ production (review [38]). We have previously shown that the AD OM cells secrete increased amounts of Aβ_1–42_ [7]. While direct evidence is still lacking, it is plausible that elevations of zinc observed in the AD OM cells can result in increased aggregation of Aβ, yet further studies are still needed.

Alpha-2-macroglobulin, A2M, (i) is found in senile plaques of AD [39], (ii) can bind Aβ with high affinity [40], and (iii) can mediate the Aβ clearance in cells [41]. Interestingly, neuronal A2M is reported to be mostly expressed in the entorhinal cortex and hippocampus [39], brain areas affected early in the disease pathogenesis along the olfactory processing areas. Furthermore, a variant of A2M (*A2M-2* allele) has been linked with elevated risk for AD [42]. In this study, we observed both the transcripts of *A2M* and the A2M protein levels to be increased in AD OM cells, supporting the previous findings of increased A2M levels in AD affected brains [14] and plasma (systematic review [15]). Interestingly, plasma A2M levels are known to correlate with cerebral amyloid burden [43] and are now considered as a component of a panel of plasma biomarkers for early AD [44]. Furthermore, A2M alterations have been previously reported in other diseases besides AD, such as chronic lung diseases [45], liver fibrosis [46] and are linked to patients with congenital antithrombin deficiency [47]. In addition, decreased serum levels of A2M have been reported in prostate cancer with bone metastases [48].

It is important to note that in the presence of zinc, the ability of A2M to bind Aβ is enhanced [40]. Upon A2M binding to Aβ, the A2M–Aβ complex can be cleared by the LDL receptor related protein 1, LRP1 [41]. We have previously shown the expression of *LRP1* to be significantly upregulated in fibroblast/stromal-like cells of the AD OM, when compared to the cognitively healthy controls [7]. In addition to the *A2M*, also variants of the other interaction partners of LRP1, for example, *APP* encoding for the amyloid precursor protein, and the gene for apolipoprotein E, *APOE*, have a well-known link to AD (review [1]). Our data suggest a link between increased A2M levels in response to increased Aβ, facilitating a potential means of clearance of A2M–Aβ complexes. The binding and degradation are affected by metals; hence, biometal homeostasis can play an important role in the process. Taken together, we consider that the impairments of biometal homeostasis in the OM can recapitulate the alterations observed in the brain. Given that the sense of smell is critical for the cognitive process of autobiographical retrieval known to be compromised in AD [49], impairments in the proper functioning of the OM could be related to memory, and thus provide a potential site for the early diagnosis of the disease.

## 4. Materials and Methods

### 4.1. Patients and Culture of OM Cells

Primary OM cell cultures from 13 cognitively healthy controls (age range 64–73 years), 11 individuals with amnestic MCI (later, MCI age range 62–82 years), and from 12 patients with mild AD (age range 58–80 years) were used in this study. Prior to harvesting the tissues from donors, diagnostic examinations were carried out at the Kuopio University Hospital, and University of Eastern Finland Brain Research Unit, Finland, as previously described [7] under the ethical approval from the Research Ethics Committee of the Northern Savo Hospital District (permit number 536/2017). All study participants underwent careful demographic interview, medical records were checked, Clinical Dementia Rating (CDR) scale evaluation [50] and the Consortium to Establish a Registry for Alzheimer’s Disease neuropsychological battery (CERAD-NB) was carried out at the screening visit. The CERAD test battery is a standardized test battery developed to measure primarily AD-related cognitive decline [51]. It is widely used and gives less false negative results than MMSE [52]. The study group (control, MCI or AD) was confirmed by the study clinicians and psychologists specialized to memory disorders. The study participants were accepted to the control group if they performed the cognitive tests within normal limits and they had no cognitive impairment or decline in daily functions on the basis of demographic and CDR interview. The participants that demonstrated reduced performance in at least one memory domain in the CERAD-NB but were normal with regard to performance in daily activities and did not meet the NIA/AA criteria for dementia formed the MCI group for this study. The AD patients were diagnosed using the revised National Institute on Aging and Alzheimer’s Association (NIA/AA) criteria [53] by neurologists. The differential diagnostic laboratory tests, and neuropsychological test battery had been performed for all of them before this study. All the AD patients had initiated appropriate AD medication. However, the stage of AD was confirmed after study evaluations. Persons with neurodegenerative disorders or MCI relating to a condition other than AD or without amnestic MCI were excluded as well as persons with moderate (CDR 2) or severe (CDR 3) AD. Furthermore, a structural brain MRI was carried out for all the MCI and AD patients if a previous brain MRI was more than 18 months older, was not available or there were new findings in the clinical screening evaluation. Persons with minor vascular changes (Fazekas 1) in brain MRI were included in the study if the other evaluations supported clinically amnestic MCI or mild AD. Written informed consent was collected from all study participants and proxy consent from the family members/legally acceptable representatives of persons with amnestic MCI or AD.

The OM cells were cultured as previously described [7,54]. Briefly, the OM cells harvested from a biopsy of the nasal septum were cultured in medium (DMEM/F12, #11320033) supplemented with 10% heat-inactivated FBS (#10270106) and 1× Penicillin–Streptomycin (#15140122), all reagents obtained from Gibco (Waltham, MA, USA), at 37 °C, 5% CO_2_. The cells used in this study were in primary passages 4–7.

### 4.2. RNA Extraction and RNA Sequencing

Total RNA of OM cells cultured at passages 4–5 was extracted using RNeasy Plus Mini Kit (Qiagen, Hilden, Germany), according to the manufacturer’s instructions with a minor adjustment of addition of β-mercaptoethanol to RLT Plus reagent. Agilent 2100 Bioanalyzer with the RNA 6000 Pico kit was used to evaluate integrity of the isolated RNA (Agilent, Santa Clara, CA, USA). Prior to RNA-seq library preparation, rRNA was depleted from 100 ng of total RNA input with QIAseq Fast Select RNA Removal Kit (Qiagen) following the manufacturer’s instructions. RNA libraries were prepared using QIAseq Stranded Total RNA Library Kit (Qiagen), according to the manufacturer’s instructions. Concentrations of the amplified libraries were measured with Qubit fluorometer and dsDNA HS assay kit (Invitrogen, Waltham, MA, USA). Library size distribution was visualized with Agilent 5200 Fragment Analyzer and HS NGS Fragment Kit (1–6000 bp). Libraries were pooled and subsequently sequenced on Illumina NovaSeq 6000 platform using S1 Reagent Kit (Illumina, San Diego, CA, USA). The 2 × 100 bp paired-end sequencing resulted in approximately 50 million reads per sample.

### 4.3. RNA Sequencing Data Processing and Analysis

For each of samples, two separate lanes were sequenced, and the resulting forward and reverse read fastq files of different lanes were merged together using the cat bash command. After lane file merging, read quality control was performed using the FastQC (v0.11.9) tool, Trimmomatic (v.0.39) [55] was used to remove potential TruSeq adapters sequences and trimmed reads were then aligned to the GENCODE [56] reference genome (GRCh38), previously indexed through a primary assembly annotation gtf file (v36), using the STAR tool (v.2.7.7) [57]. The FeatureCounts (v.2.0.0) [58] tool of the package Subread [59] was used to quantify the mapped reads and to achieve the raw count matrix.

Downstream and statistical analyses were carried out using the R package DESeq2 [60] of the Bioconductor (review [61]) framework. Genes not expressed in at least one sample for each sample’s condition (AD, MCI, Controls) and with a total number of reads mapped in all the samples lower than 10 were filtered out. PCA plots, used to identify potential sources of variation inside the dataset, showed a batch effect due to the sample sequencing group; therefore, the batch variable was used as a covariate in the statistical model together with the sample condition and other biological information including gender, *APOE* genotype and the age, previously categorized in seven groups using the R function cut(), of the samples. Before proceeding with the differential expression analysis, sample AD1, appearing as an outlier from PCA plots, was removed from the dataset. Differential expression analysis was performed using the DESeq() function and significantly differentially expressed genes between Alzheimer’s disease control and MCI samples, with an adjusted *p*-value lower than 0.1, were retrieved using the lfcShrink() function excluding non-converging ones and shrinking the LogFoldChanges using the “ashr“ method [62].

Functional analysis of the significantly differentially expressed genes was performed. Genes were used as input for the enrichment R package gprofiler2 [63] and the analysis was conducted querying different databases including Gene Ontology [64,65] and CORUM [66].

To further explore biological functions perturbed, enrichment analysis was also performed through an active subnetwork enrichment method called pathfindR [16] and using Gene Ontology gene sets [64,65].

### 4.4. Protein Extraction and Western Blotting

OM cells (primary passages 4–7) were plated at an approximate density of 15000 cells per cm^2^ on 6-well culture plates and kept in culture for 96 h prior to collection for Western blot. First, cells were washed with D-PBS (Gibco, Waltham, MA, USA); then scraped and collected into 1× Laemmli buffer for storage at −20 °C. Total protein measurement was performed using Pierce 660nm Protein Assay Reagent (Thermo Fisher, Waltham, MA, USA) according to the commercial protocol. Samples (20 µg of protein) were denaturated at 95 °C for 5 min and separated by electrophoresis on 7.5% polyacrylamide gels. Proteins were transferred to poly(vinylidene fluoride) (GE Healthcare, Chicago, IL, USA) membranes by semi-dry blotting. The membranes were blocked in 5% nonfat dry milk in phosphate-buffered saline with Tween-20 (1× PBS, 7.4 pH, 0.2% Tween) and incubated overnight with primary antibodies at 4 °C: β-actin (1:5000, A5441, Sigma-Aldrich, St. Louis, MO, USA) and α2M (1:1000, 13545-1-AP, Proteintech Group Inc., Rosemont, IL, USA). The signals were detected with Cy5 conjugated AffiniPure Donkey Anti-Mouse IgG (H+L) (1:1000, Jackson Immuno Research Laboratories Europe Ltd., Cambridgeshire, UK) for β-actin (incubated for 2 h, at RT, protected from light); and anti-rabbit horseradish peroxidase-conjugated antibody (1:3000, 130-65-15, BioRad, Hercules, CA, USA) for α2M, using a substrate solution (Pierce SuperSignal West Pico Plus Chemiluminescent substrate, Waltham, MA, USA) for signal enhancement. For the imaging, a Bio-Rad ChemiDoc XRS+ System was used and the analysis of the blots was performed with Image Lab (6.0) software (Bio-Rad, Hercules, CA, USA).

### 4.5. Metal Quantitation via ICP-MS

Cells for ICP-MS were collected as dry cell pellets from a total of 11 OM cell lines derived from cognitively healthy controls and 10 patients with AD at passages 4–6. Working on ice, the cell culture medium from a total of three 6-wells/line was replaced with 1× tris-buffered saline (TBS) and cells from three wells were scraped in 1× TBS to collect the cells. Next, the cells were pelleted by centrifugation for 720× *g* for 3 min and the resulting soft pellet was resuspended in 1 mL of 1× TBS. An aliquot of the cell suspension was used for protein quantification and the remaining cell suspension was centrifuged at 1500× *g* for 3 min to pellet the cells. The samples were stored as dry cell pellets at −70 °C prior to processing for the ICP-MS.

Next, 30 µL of concentrated nitric acid (65%, Suprapur, Merck, Darmstadt, Germany) was added to each cell pellet and allowed to digest overnight at room temperature. Then, the samples were heated at 90 °C for 20 min in a heat block to complete the digestion. A total of 470 µL of 1% nitric acid (*v*/*v*) was added to each sample to reach a total volume of 500 µL, and the samples were stored at 4 °C until the day of the analysis. Measurements were made using an Agilent 7700 series ICP-MS instrument under routine multi-element operating conditions using a Helium Reaction Gas Cell as previously described [67].

Protein concentration of the cell samples were measured with a commercial kit based on a colorimetric quantification of the total protein according to the manufacturer’s instructions (Pierce™ BCA Protein Assay Kit (Thermo Scientific, Waltham, MA, USA, #23227)). The results were normalized to the protein concentration of the cell samples and calculated as µmol/µg protein ((raw ppb value x final sample volume/molecular weight of the element)/total protein in the sample).

### 4.6. Statistical Analysis

GraphPad Prism 8.1.0 (GraphPad Software Inc., San Diego, CA, USA) software was used for statistical analysis of the data. Mean values in ICP-MS and Western blot analyses were compared using an unpaired two-tailed *t*-test. Error bars in the figure legends represent standard deviation (SD). Statistical outliers were removed for each assay with iterative Grubb’s method (alpha 0.1 for ICP-MS and alpha 0.05 for Western blot). Statistical significance was assumed for adjusted *p*-value < 0.1 for the RNA sequencing data and for *p*-values ≤ 0.05 for the Western blot and ICP-MS.

## 5. Conclusions

This study provides new insight into biometal alterations in cells of the olfactory mucosa in AD. In line with published literature on the brain, we report AD-associated alterations to metal levels and metal-related genes in cells of the OM, further providing support for the notion that these cells show changes related to the disease processes.

## Figures and Tables

**Table 1:** Differentially expressed genes (DEGs) with metal-ion binding abilities or other metal-related function between the Alzheimer’s disease (AD) and control olfactory mucosa (OM) cells identified by total RNA sequencing. FC, fold change. padj-value (adjusted *p*-value, false discovery rate) < 0.1.

Gene Symbol	Gene Name	Log2FC	*p*-Value	padj-Value	Metal-Binding/Metal-Related Function ^1^
*NOTCH4*	notch receptor 4	6.31	2.72 × 10^−6^	0.0110	metal-binding; Ca
*LMTK3*	lemur tyrosine kinase 3	6.29	3.90 × 10^−6^-	0.0127	metal-binding; Mg
*ATP8A2*	ATPase phospholipid transporting 8A2	4.49	4.19 × 10^−7^	0.0034	metal-binding; Mg
*KDM4A-AS1*	KDM4A antisense RNA 1	4.30	1.85 × 10^−5^	0.0251	for KDM4A: metal-binding; Fe, Zn
*ADGRE2*	adhesion G protein-coupled receptor E2	3.27	1.45 × 10^−6^	0.0079	metal-binding; Ca
*NKD1*	NKD inhibitor of WNT signaling pathway 1	2.78	9.71 × 10^−5^	0.0533	metal-binding; Ca
*DCHS1*	dachsous cadherin-related 1	2.39	8.83 × 10^−6^	0.0211	metal-binding; Ca
*A2M*	alpha-2-macroglobulin	2.26	1.02 × 10^−4^	0.0533	Ca-dependent protein binding
*ZNF501*	zinc finger protein 501	2.06	1.05 × 10^−5^	0.0212	metal-binding; Zn
*PRDM1*	PR/SET domain 1	1.96	3.17 × 10^−7^	0.0034	metal-binding; Zn
*PLS1*	plastin 1	1.92	1.52 × 10^−4^	0.0686	metal-binding; Ca
*PHF7*	PHD finger protein 7	1.90	7.37 × 10^−5^	0.0460	metal-binding; Zn
*CORO1A*	coronin 1A	1.67	2.66 × 10^−4^	0.0938	calcium ion transport
*S100A3*	S100 calcium binding protein A3	1.28	1.68 × 10^−4^	0.0698	metal-binding; Ca, Zn
*RNF24*	ring finger protein 24	0.17	1.39 × 10^−4^	0.0644	metal-binding; Zn
*THBS3*	thrombospondin 3	−0.34	1.33 × 10^−4^	0.0635	calcium ion binding
*RIMS1*	regulating synaptic membrane exocytosis 1	−2.94	5.49 × 10^−5^	0.0397	metal-binding; Zn

^1^ UniProt (https://www.uniprot.org/, accessed on 16 February 2022).

**Table 2:** Differentially expressed genes (DEGs) with metal-ion binding abilities or other metal-related function between the mild cognitive impairment (MCI) and control olfactory mucosa (OM) cells identified by total RNA sequencing. FC, fold change. padj-value (adjusted *p*-value, false discovery rate) < 0.1.

Gene Symbol	Gene Name	Log2FC	*p*-Value	padj Value	Metal-Binding/Metal-Related Function ^1^
*GNG4*	G protein subunit gamma 4	4.42	2.71 × 10^−6^	0.0337	pathway, Ca
*NOTCH4*	notch receptor 4	3.66	8.31 × 10^−6^	0.0337	metal-binding; Ca
*SYTL5*	synaptotagmin like 5	3.25	1.27 × 10^−5^	0.0339	metal-binding; Zn
*A2M*	alpha-2-macroglobulin	2.45	1.47 × 10^−5^	0.0339	Ca-dependent protein binding
*STC1*	stanniocalcin 1	1.16	2.94 × 10^−5^	0.0397	Ca homeostasis
*PRDM1*	PR/SET domain	1.02	1.07 × 10^−5^	0.0339	metal-binding; Zn
*HTR2B*	5-hydroxytryptamine receptor 2B	1.00	1.24 × 10^−4^	0.0856	Ca-mediated signaling
*ZNF408*	zinc finger protein 408	0.85	6.13 × 10^−6^	0.0337	metal-binding; Zn
*CHURC1*	churchill domain containing 1	0.72	3.49 × 10^−5^	0.0435	metal-binding; Zn
*SLC46A1*	solute carrier family 46 member 1	0.45	2.66 × 10^−5^	0.0392	iron homeostasis
*DSE*	dermatan sulfate epimerase	−0.10	6.20 × 10^−5^	0.0628	metal-binding, Mn
*ENPP5*	ectonucleotide pyrophosphatase/phosphodiesterase family member 5	−1.17	1.27 × 10^−4^	0.0856	metal-binding; Zn
*ADAMTS14*	ADAM metallopeptidase with thrombospondin type 1 motif 14	−1.21	9.42 × 10^−5^	0.0727	metal-binding; Zn
*AGBL2*	AGBL carboxypeptidase 2	−1.77	7.85 × 10^−5^	0.0706	metal-binding; Zn

^1^ UniProt (https://www.uniprot.org/, accessed on 16 February 2022).

**Table 3:** The elemental content of Na, Mg, P, K, Ca, Mn, Fe, Cu and Zn in Alzheimer’s disease (AD) and control olfactory mucosa (OM) cells measured by inductively coupled plasma mass spectrometry (ICP-MS).

Metal	Controls Mean ± SEM ^†^	AD Mean ± SEM ^†^	Unpaired *t*-Test (Two-Tailed) *p*-Value
^23^Na	7436 ± 450.7	11,600 ± 1005	0.0020 *
^24^Mg	13.29 ± 1.627	16.16 ± 1.553	0.2252
^31^P	543.4 ± 45.32	629.9 ± 37.82	0.1716
^39^K	6.645 ± 0.7977	7.674 ± 0.5793	0.3181
^43^Ca	7.299 ± 0.754	10.02 ± 1.008	0.0416 *
^55^Mn	0.1171 ± 0.01115	0.1437 ± 0.01158	0.1153
^56^Fe	1.718 ± 0.1663	2.047 ± 0.2251	0.2499
^63^Cu	0.1836 ± 0.02722	0.3156 ± 0.08026	0.1218
^66^Zn	1.899 ± 0.1417	2.385 ± 0.1570	0.0340 *

^†^ Elemental content normalized to total protein level and shown as µmol/µg protein. Outliers within the data groups were tested with iterative Grubb’s method (alpha 0.1). Unpaired two-tailed *t*-test. * *p* ≤ 0.05.

## Data Availability

The data presented in this study are available on request from the corresponding author. RNA sequencing data are available from the European Genome-phenome Archive (EGA, https://ega-archive.org/, 7 February 2022) under the accession ID EGAD00001008707.

## Supplementary Materials

The following supporting information can be downloaded at: https://www.mdpi.com/article/10.3390/ijms23084123/s1.

## Appendix A

**Table A1:** Demographic information of the biopsy donors of olfactory mucosa (OM) cell lines used for RNA sequencing.

Study Group	*n*	Age *	Sex	Other Diseases
Controls	10	71.1 ± 3.8	Females 70.0% Males 30.0%	Cardiovascular 100.0% Thyroid 30.0% Respiratory 10.0% Autoimmune 20.0% Diabetes 0.0%
AD	12	68.3 ± 7.4	Females 50.0% Males 50.0%	Cardiovascular 66.7% Thyroid 8.3% Respiratory 0.0% Autoimmune 0.0% Diabetes 33.3%
MCI	11	70.9 ± 6.4	Females 45.5% Males 54.5%	Cardiovascular 63.6% Thyroid 9.1% Respiratory 9.1% Autoimmune 27.3% Diabetes 18.2%

*** Mean ± SD.

**Table A2:** Demographic information of the biopsy donors of olfactory mucosa (OM) cell lines used for inductively coupled plasma mass spectrometry (ICP-MS).

Study Group	*n*	Age *	Sex
Controls	11	69.1 ± 3.2	Females 63.6%
			Males 36.4%
AD	10	68.7 ± 7.7	Females 60.0%
			Males 40.0%

*** Mean ± SD.

## References

[1] Scheltens P., de Strooper B., Kivipelto M., Holstege H., Chételat G., Teunissen C.E., Cummings J., van der Flier W.M. (2021). Alzheimer’s Disease. Lancet.

[2] Lane C.A., Hardy J., Schott J.M. (2018). Alzheimer’s Disease. Eur. J. Neurol..

[3] Jack C.R., Knopman D.S., Jagust W.J., Shaw L.M., Aisen P.S., Weiner M.W., Petersen R.C., Trojanowski J.Q. (2010). Hypothetical Model of Dynamic Biomarkers of the Alzheimer’s Pathological Cascade. Lancet Neurol..

[4] Sohrabi H.R., Bates K.A., Rodrigues M., Taddei K., Laws S.M., Lautenschlager N.T., Dhaliwal S.S., Johnston A.N.B., MacKay-Sim A., Gandy S. (2009). Olfactory Dysfunction Is Associated with Subjective Memory Complaints in Community-Dwelling Elderly Individuals. J. Alzheimers Dis..

[5] Sohrabi H.R., Bates K.A., Weinborn M.G., Johnston A.N.B., Bahramian A., Taddei K., Laws S.M., Rodrigues M., Morici M., Howard M. (2012). Olfactory Discrimination Predicts Cognitive Decline among Community-Dwelling Older Adults. Transl. Psychiatry.

[6] Jung H.J., Shin I.S., Lee J.E. (2019). Olfactory Function in Mild Cognitive Impairment and Alzheimer’s Disease: A Meta-Analysis. Laryngoscope.

[7] Lampinen R., Feroze Fazaludeen M., Avesani S., Örd T., Penttilä E., Lehtola J.-M., Saari T., Hannonen S., Saveleva L., Kaartinen E. (2022). Single-Cell RNA-Seq Analysis of Olfactory Mucosal Cells of Alzheimer’s Disease Patients. Cells.

[8] Fasae K.D., Abolaji A.O., Faloye T.R., Odunsi A.Y., Oyetayo B.O., Enya J.I., Rotimi J.A., Akinyemi R.O., Whitworth A.J., Aschner M. (2021). Metallobiology and Therapeutic Chelation of Biometals (Copper, Zinc and Iron) in Alzheimer’s Disease: Limitations, and Current and Future Perspectives. J. Trace Elem. Med. Biol..

[9] Samudralwar D.L., Diprete C.C., Ni B.F., Ehmann W.D., Markesbery W.R. (1995). Elemental Imbalances in the Olfactory Pathway in Alzheimer’s Disease. J. Neurol. Sci..

[10] Ono S.I., Cherian G.M. (1999). Regional Distribution of Metallothionein, Zinc, and Copper in the Brain of Different Strains of Rats. Biol. Trace Elem. Res..

[11] Sastre M., Ritchie C.W., Hajji N. (2015). Metal Ions in Alzheimer’s Disease Brain. JSM Alzheimers Dis. Related Dementia.

[12] Tamano H., Takeda A. (2019). Age-Dependent Modification of Intracellular Zn^2+^ Buffering in the Hippocampus and Its Impact. Biol. Pharm. Bull..

[13] Zaręba N., Kepinska M. (2020). The Function of Transthyretin Complexes with Metallothionein in Alzheimer’s Disease. Int. J. Mol. Sci..

[14] Wood J.A., Wood P.L., Ryan R., Graff-Radford N.R., Pilapil C., Robitaille Y., Quirion R. (1993). Cytokine Indices in Alzheimer’s Temporal Cortex: No Changes in Mature IL-1 Beta or IL-1RA but Increases in the Associated Acute Phase Proteins IL-6, Alpha 2-Macroglobulin and C-Reactive Protein. Brain Res..

[15] Kiddle S.J., Sattlecker M., Proitsi P., Simmons A., Westman E., Bazenet C., Nelson S.K., Williams S., Hodges A., Johnston C. (2014). Candidate Blood Proteome Markers of Alzheimer’s Disease Onset and Progression: A Systematic Review and Replication Study. J. Alzheimers Dis..

[16] Ulgen E., Ozisik O., Sezerman O.U. (2019). PathfindR: An R Package for Comprehensive Identification of Enriched Pathways in Omics Data through Active Subnetworks. Front. Genet..

[17] Shibata N., Ohnuma T., Higashi S., Higashi M., Usui C., Ohkubo T., Watanabe T., Kawashima R., Kitajima A., Ueki A. (2007). Genetic Association between Notch4 Polymorphisms and Alzheimer’s Disease in the Japanese Population. J. Gerontol. A Biol. Sci. Med. Sci..

[18] Kapoor A., Nation D.A. (2021). Role of Notch Signaling in Neurovascular Aging and Alzheimer’s Disease. Semin. Cell Dev. Biol..

[19] Seyfried N.T., Dammer E.B., Swarup V., Geschwind D.H., Lah J.J., Levey A.I. (2017). A Multi-Network Approach Identifies Protein-Specific Co-Expression in Asymptomatic and Symptomatic Alzheimer’s Disease. Cell Syst..

[20] Kim Y.H., Beak S.H., Charidimou A., Song M. (2016). Discovering New Genes in the Pathways of Common Sporadic Neurodegenerative Diseases: A Bioinformatics Approach. J. Alzheimers Dis..

[21] Hondius D.C., van Nierop P., Li K.W., Hoozemans J.J.M., van der Schors R.C., van Haastert E.S., van der Vies S.M., Rozemuller A.J.M., Smit A.B. (2016). Profiling the Human Hippocampal Proteome at All Pathologic Stages of Alzheimer’s Disease. Alzheimers Dement..

[22] Huat T.J., Camats-Perna J., Newcombe E.A., Valmas N., Kitazawa M., Medeiros R. (2019). Metal Toxicity Links to Alzheimer’s Disease and Neuroinflammation. J. Mol. Biol..

[23] Burnet F.M. (1981). A Possible Role of Zinc in the Pathology of Dementia. Lancet.

[24] Witt B., Schaumlöffel D., Schwerdtle T. (2020). Subcellular Localization of Copper-Cellular Bioimaging with Focus on Neurological Disorders. Int. J. Mol. Sci..

[25] Lindeque J.Z., Levanets O., Louw R., van der Westhuizen F.H. (2010). The Involvement of Metallothioneins in Mitochondrial Function and Disease. Curr. Protein Pept. Sci..

[26] Huiliang Z., Mengzhe Y., Xiaochuan W., Hui W., Min D., Mengqi W., Jianzhi W., Zhongshan C., Caixia P., Rong L. (2021). Zinc Induces Reactive Astrogliosis through ERK-Dependent Activation of Stat3 and Promotes Synaptic Degeneration. J. Neurochem..

[27] Lengyel I., Flinn J.M., Peto T., Linkous D.H., Cano K., Bird A.C., Lanzirotti A., Frederickson C.J., van Kuijk F.J.G.M. (2007). High Concentration of Zinc in Sub-Retinal Pigment Epithelial Deposits. Exp. Eye Res..

[28] Kaarniranta K., Salminen A., Haapasalo A., Soininen H., Hiltunen M. (2011). Age-Related Macular Degeneration (AMD): Alzheimer’s Disease in the Eye?. J. Alzheimers Dis..

[29] LaFerla F.M. (2002). Calcium Dyshomeostasis and Intracellular Signalling in Alzheimer’s Disease. Nat. Rev. Neurosci..

[30] Block E., Batista V.S., Matsunami H., Zhuang H., Ahmed L. (2017). The Role of Metals in Mammalian Olfaction of Low Molecular Weight Organosulfur Compounds. Nat. Prod. Rep..

[31] Sunderman F.W. (2001). Nasal Toxicity, Carcinogenicity, and Olfactory Uptake of Metals. Ann. Clin. Lab. Sci..

[32] McBride K., Slotnick B., Margolis F.L. (2003). Does Intranasal Application of Zinc Sulfate Produce Anosmia in the Mouse? An Olfactometric and Anatomical Study. Chem. Senses.

[33] Davidson T.M., Smith W.M. (2010). The Bradford Hill Criteria and Zinc-Induced Anosmia: A Causality Analysis. Arch. Otolaryngol. Head Neck Surg..

[34] Whitcroft K.L., Ezzat M., Cuevas M., Andrews P., Hummel T. (2017). The Effect of Intranasal Sodium Citrate on Olfaction in Post-Infectious Loss: Results from a Prospective, Placebo-Controlled Trial in 49 Patients. Clin. Otolaryngol..

[35] Bush A.I., Pettingell W.H., Multhaup G., Paradis M.D., Vonsattel J.P., Gusella J.F., Beyreuther K., Masters C.L., Tanzi R.E. (1994). Rapid Induction of Alzheimer A Beta Amyloid Formation by Zinc. Science.

[36] Wang L., Yin Y.L., Liu X.Z., Shen P., Zheng Y.G., Lan X.R., Lu C.B., Wang J.Z. (2020). Current Understanding of Metal Ions in the Pathogenesis of Alzheimer’s Disease. Transl. Neurodegener..

[37] Wang C.Y., Wang T., Zheng W., Zhao B.L., Danscher G., Chen Y.H., Wang Z.Y. (2010). Zinc Overload Enhances APP Cleavage and Aβ Deposition in the Alzheimer Mouse Brain. PLoS ONE.

[38] Ryan K.C., Ashkavand Z., Norman K.R. (2020). The Role of Mitochondrial Calcium Homeostasis in Alzheimer’s and Related Diseases. Int. J. Mol. Sci..

[39] Tooyama I., Kawamata T., Akiyama H., Moestrup S.K., Gliemann J., McGeer P.L. (1993). Immunohistochemical Study of Alpha 2 Macroglobulin Receptor in Alzheimer and Control Postmortem Human Brain. Mol. Chem. Neuropathol..

[40] Du Y., Ni B., Glinn M., Dodel R.C., Bales K.R., Zhang Z., Hyslop P.A., Paul S.M. (1997). Alpha2-Macroglobulin as a Beta-Amyloid Peptide-Binding Plasma Protein. J. Neurochem..

[41] Narita M., Holtzman D.M., Schwartz A.L., Bu G. (1997). Alpha2-Macroglobulin Complexes with and Mediates the Endocytosis of Beta-Amyloid Peptide via Cell Surface Low-Density Lipoprotein Receptor-Related Protein. J. Neurochem..

[42] Blacker D., Wilcox M.A., Laird N.M., Rodes L., Horvath S.M., Go R.C.P., Perry R., Watson B., Bassett S.S., McInnis M.G. (1998). Alpha-2 Macroglobulin Is Genetically Associated with Alzheimer Disease. Nat. Genet..

[43] Westwood S., Leoni E., Hye A., Lynham S., Khondoker M.R., Ashton N.J., Kiddle S.J., Baird A.L., Sainz-Fuertes R., Leung R. (2016). Blood-Based Biomarker Candidates of Cerebral Amyloid Using PiB PET in Non-Demented Elderly. J. Alzheimers Dis..

[44] Eke C.S., Jammeh E., Li X., Carroll C., Pearson S., Ifeachor E. (2021). Early Detection of Alzheimer’s Disease with Blood Plasma Proteins Using Support Vector Machines. IEEE J. Biomed. Health Inform..

[45] Poller W., Barth J., Voss B. (1989). Detection of an Alteration of the Alpha 2-Macroglobulin Gene in a Patient with Chronic Lung Disease and Serum Alpha 2-Macroglobulin Deficiency. Hum. Genet..

[46] Ho A.S., Cheng C.C., Lee S.C., Liu M.L., Lee J.Y., Wang W.M., Wang C.C. (2010). Novel Biomarkers Predict Liver Fibrosis in Hepatitis C Patients: Alpha 2 Macroglobulin, Vitamin D Binding Protein and Apolipoprotein AI. J. Biomed. Sci..

[47] Tripodi A., Chantarangkul V., de Stefano V., Mannucci P. (2000). Alpha(2)-Macroglobulin Levels Are High in Adult Patients with Congenital Antithrombin Deficiency. Thromb. Res..

[48] Kanoh Y., Ohtani N., Mashiko T., Ohtani S., Nishikawa T., Egawa S., Baba S., Ohtani H. (2001). Levels of Alpha 2 Macroglobulin Can Predict Bone Metastases in Prostate Cancer. Anticancer Res..

[49] El Haj M., Gandolphe M.C., Gallouj K., Kapogiannis D., Antoine P. (2018). From Nose to Memory: The Involuntary Nature of Odor-Evoked Autobiographical Memories in Alzheimer’s Disease. Chem. Senses.

[50] O’Bryant S.E., Lacritz L.H., Hall J., Waring S.C., Chan W., Khodr Z.G., Massman P.J., Hobson V., Cullum C.M. (2010). Validation of the New Interpretive Guidelines for the Clinical Dementia Rating Scale Sum of Boxes Score in the National Alzheimer’s Coordinating Center Database. Arch. Neurol..

[51] Welsh K.A., Butters N., Hughes J.P., Mohs R.C., Heyman A. (1992). Detection and Staging of Dementia in Alzheimer’s Disease. Use of the Neuropsychological Measures Developed for the Consortium to Establish a Registry for Alzheimer’s Disease. Arch. Neurol..

[52] Chandler M.J., Lacritz L.H., Hynan L.S., Barnard H.D., Allen G., Deschner M., Weiner M.F., Cullum C.M. (2005). A Total Score for the CERAD Neuropsychological Battery. Neurology.

[53] McKhann G.M., Knopman D.S., Chertkow H., Hyman B.T., Jack C.R., Kawas C.H., Klunk W.E., Koroshetz W.J., Manly J.J., Mayeux R. (2011). The Diagnosis of Dementia Due to Alzheimer’s Disease: Recommendations from the National Institute on Aging-Alzheimer’s Association Workgroups on Diagnostic Guidelines for Alzheimer’s Disease. Alzheimers Dement..

[54] Chew S., Lampinen R., Saveleva L., Korhonen P., Mikhailov N., Grubman A., Grubman A., Grubman A., Polo J.M., Polo J.M. (2020). Urban Air Particulate Matter Induces Mitochondrial Dysfunction in Human Olfactory Mucosal Cells. Part. Fibre Toxicol..

[55] Bolger A.M., Lohse M., Usadel B. (2014). Trimmomatic: A Flexible Trimmer for Illumina Sequence Data. Bioinformatics.

[56] Frankish A., Diekhans M., Jungreis I., Lagarde J., Loveland J.E., Mudge J.M., Sisu C., Wright J.C., Armstrong J., Barnes I. (2021). GENCODE 2021. Nucleic Acids Res..

[57] Dobin A., Davis C.A., Schlesinger F., Drenkow J., Zaleski C., Jha S., Batut P., Chaisson M., Gingeras T.R. (2013). STAR: Ultrafast Universal RNA-Seq Aligner. Bioinformatics.

[58] Liao Y., Smyth G.K., Shi W. (2014). FeatureCounts: An Efficient General Purpose Program for Assigning Sequence Reads to Genomic Features. Bioinformatics.

[59] Liao Y., Smyth G.K., Shi W. (2013). The Subread Aligner: Fast, Accurate and Scalable Read Mapping by Seed-and-Vote. Nucleic Acids Res..

[60] Love M.I., Huber W., Anders S. (2014). Moderated Estimation of Fold Change and Dispersion for RNA-Seq Data with DESeq2. Genome Biol..

[61] Huber W., Carey V.J., Gentleman R., Anders S., Carlson M., Carvalho B.S., Bravo H.C., Davis S., Gatto L., Girke T. (2015). Orchestrating High-Throughput Genomic Analysis with Bioconductor. Nat. Methods.

[62] Stephens M. (2017). False Discovery Rates: A New Deal. Biostatistics.

[63] Raudvere U., Kolberg L., Kuzmin I., Arak T., Adler P., Peterson H., Vilo J. (2019). G:Profiler: A Web Server for Functional Enrichment Analysis and Conversions of Gene Lists (2019 Update). Nucleic Acids Res..

[64] Ashburner M., Ball C.A., Blake J.A., Botstein D., Butler H., Cherry J.M., Davis A.P., Dolinski K., Dwight S.S., Eppig J.T. (2000). Gene Ontology: Tool for the Unification of Biology. The Gene Ontology Consortium. Nat. Genet..

[65] Carbon S., Douglass E., Good B.M., Unni D.R., Harris N.L., Mungall C.J., Basu S., Chisholm R.L., Dodson R.J., Hartline E. (2021). The Gene Ontology Resource: Enriching a GOld Mine. Nucleic Acids Res..

[66] Giurgiu M., Reinhard J., Brauner B., Dunger-Kaltenbach I., Fobo G., Frishman G., Montrone C., Ruepp A. (2019). CORUM: The Comprehensive Resource of Mammalian Protein Complexes-2019. Nucleic Acids Res..

[67] Kanninen K.M., Grubman A., Meyerowitz J., Duncan C., Tan J.L., Parker S.J., Crouch P.J., Paterson B.M., Hickey J.L., Donnelly P.S. (2013). Increased Zinc and Manganese in Parallel with Neurodegeneration, Synaptic Protein Changes and Activation of Akt/GSK3 Signaling in Ovine CLN6 Neuronal Ceroid Lipofuscinosis. PLoS ONE.

